# Possible Role of Intestinal Fatty Acid Oxidation in the Eating-Inhibitory Effect of the PPAR-α Agonist Wy-14643 in High-Fat Diet Fed Rats

**DOI:** 10.1371/journal.pone.0074869

**Published:** 2013-09-17

**Authors:** Elnaz Karimian Azari, Claudia Leitner, Thomas Jaggi, Wolfgang Langhans, Abdelhak Mansouri

**Affiliations:** Physiology and Behavior Laboratory, ETH Zurich, Schwerzenbach, Switzerland; Sapienza University of Rome, Italy

## Abstract

PPAR-α plays a key role in lipid metabolism; it enhances fatty acid oxidation (FAO) and ketogenesis. Pharmacological PPAR-α activation improves insulin sensitivity and reduces food intake, but its mechanisms of action remain unknown. We here report that intraperitoneal (IP) administration of the PPAR-α agonist Wy-14643 (40 mg/kg BW) reduced food intake in adult male rats fed a high-fat diet (HFD, 49% of the energy) mainly through an increase in the latency to eat after injection, and without inducing a conditioned taste avoidance. Also, IP administered Wy-14643 caused an acute (the first 60 min) decrease in the respiratory quotient (RQ) and an increase in hepatic portal vein β-hydroxybutyrate level (at 35 min) without affecting plasma non-esterified fatty acids. Given the known stimulatory effect of PPAR-α on FAO and ketogenesis, we measured the protein expression level of carnitine palmitoyltransferase-1 (CPT 1A) and mitochondrial 3-hydroxy-3-methylglutaryl-coenzyme A synthase (HMG-CoAS2), two key enzymes for FAO and ketogenesis, respectively, in liver, duodenum and jejunum. Wy-14643 induced a significant increase in the expression of CPT 1A in the jejunum and duodenum and of HMG-CoAS2 in the jejunum, but neither CPT 1A nor HMG-CoAS2 expression was increased in the liver. The induction of CPT 1A and HMG-CoAS2 expression was associated with a decrease in the lipid droplet content selectively in the jejunum. Our findings indicate that Wy-14643 stimulates FAO and ketogenesis in the intestine, in particular in the jejunum, rather than in the liver, thus supporting the hypothesis that PPAR-α activation inhibits eating by stimulating intestinal FAO.

## Introduction

Obesity is the fastest growing global health threat, promoting diseases such as stroke, cardiovascular disease, type-II-diabetes mellitus and certain types of cancer [1]. Obesity develops when energy intake chronically exceeds energy expenditure. In the United States the average increase in energy intake between 1970 and 2000 appeared to be sufficient to explain the observed increase in average body weight [2]. It is therefore crucial to understand the mechanisms underlying the control of eating in order to identify possible treatment options for obesity.

The control of eating in mammals involves the complex interaction of several mechanisms that jointly initiate and terminate individual meals [3,4]. The homeostatic function of energy intake indicates that metabolic signals contribute to the control of eating, and several lines of evidence suggest that peripheral glucose utilization and fatty acid oxidation (FAO) can generate such metabolic signals [5-8]. As far as FAO is concerned, there are numerous reports of an association between an inhibition of FAO and a stimulation of eating (see 5,7). In contrast, findings of a decrease in food intake in response to a stimulation of FAO are sparse [8]. One of these examples is the activation of the peroxisome proliferator-activated receptor-alpha (PPAR-α) that increases FAO and inhibits eating in rodents [9].

PPAR-α is a nuclear hormone receptor that activates the expression of target genes by binding to their promoters, where they recognize a specific sequence of nucleotides called peroxisome proliferator response elements (PPRE). PPAR-α-regulated genes modulate important metabolic pathways such as lipolysis, FAO, and ketogenesis [10]. In fact, PPAR-α plays a crucial role in the three FAO pathways, i.e., mitochondrial and peroxisomal β-oxidation, and microsomal ω-oxidation [10]. PPAR-α increases the expression of a wide range of enzymes that promote FAO (e.g., acyl-CoA oxidase, carnitine palmitoyltransferase-1A (CPT 1), malonyl-CoA decarboxylase), and down-regulates enzymes involved in *de novo* fatty acid synthesis [11-13]. Fatty acid-binding proteins, FAT/CD36 and fatty acid transport proteins, which participate in the cellular uptake of fatty acids, are up-regulated by endogenous and synthetic PPAR-α agonists such as oleylethanolamide (OEA) and fenofibrates and appear to be necessary for these compounds’ eating-inhibitory effects [9,14]. Dietary long-chain saturated and unsaturated fatty acids, both, and fatty acid catabolism derivatives, can activate PPAR-α [10]. It has been recently shown that elaidyl-sulfamide (ES), a sulfamolyl OEA analogue, also activates PPAR-α and induces a potent reduction in food intake [15]. Moreover, changes in the expression level of PPAR-α correspond to changes in the nutritional status. Exposure to high fat diets and food deprivation enhance the PPAR-α-dependent signaling in liver and intestine [16]. In general, PPAR-α is expressed in a variety of tissues with high energy demand, including liver, kidney, muscle, brown adipose tissue, heart [17], and small intestine [14,17-20].

Intraperitoneally administered pirinixic acid (Wy-14643), a synthetic PPAR-α agonist [21], reduced food intake in rats [9] and mice [22]. Some findings suggest that gastrointestinal sensory fibers are required for the eating-inhibitory effect of OEA [23,24], a potent PPAR-α agonist. On the other hand, the eating-inhibitory effect of OEA appears to be mediated through the central release of oxytocin [25]. Nevertheless, the exact mechanisms through which PPAR-α agonists reduce food intake are still elusive. Therefore, the goal of this study was to further investigate the effect of Wy-14643 on eating behavior and to start characterizing the possible mechanism of its hypophagic effect. We show that intraperitoneal (IP) injections of Wy-14643 (40 mg/kg body weight = BW) reduced food intake in rats fed a high-fat diet without inducing avoidance and mainly by reducing the latency to eat. Also, Wy-14643 decreased the respiratory quotient (RQ), indicating an increase in whole body FAO. Further, Wy-14643 induced the protein expression of key enzymes involved in FAO and ketogenesis, such as CPT 1A and mitochondrial 3-hydroxy-3-methylglutaryl-CoA synthase (HMG-CoAS2) in the small intestine, but not in the liver. This, together with the observed increase in hepatic portal vein β-hydroxbutyrate (BHB) despite a lack of change in circulating non-esterified fatty acid (NEFA) levels, and a decrease in lipid droplets in the jejunum suggest that the hypophagic effect of Wy-14643 in high-fat diet-fed rats is mediated by an increase in intestinal FAO.

## Materials and Methods

### Ethics Statement

The Veterinary Office of the Canton of Zurich (Switzerland) approved all the animal protocols reported in this study.

### Animals and housing

Male Sprague Dawley rats (Charles Rivers, Sulzfeld, Germany) weighing 180–200 g upon arrival, were individually housed in custom-made acrylic infusion cages [21 × 37 × 41 cm, length (l) × width (w) × height (h)] with stainless steel grid floors and kept under a 12: 12 h dark/light cycle (lights off 0800 h) in a climate-controlled room (22 ± 2 °C and 60% relative humidity). The rats had *ad libitum* access to tap water and a fat-enriched diet (HFD, 49% kcal from fat, No. D10022601M, Research Diets Inc., New Brunswick, NJ, USA; n = 37) unless otherwise noted. We used this diet because high fat diets supposedly increase PPAR-α-dependent signaling [10]. Food cups placed on balances (XS4001S, Mettler-Toledo, Greifensee, Switzerland) were accessible through a niche (5 x 7 x 30 cm, 6 cm above the cage floor) on one side of the cage, and a radio tuned to a music station was played continuously to mask external noise. The balances were connected to a computer with a custom-designed software allowing for meal pattern analysis [26]. The animals were adapted to housing and diet conditions for at least 2 weeks prior to the start of the experiment.

### Catheters and implantation

Rats were equipped with intraperitoneal (IP, n = 66) and hepatic portal vein (HPV, n = 16) catheters for substance administration and blood samplings, respectively. Catheters were sterilized with ethylene oxide before use and implanted using sterile techniques. The custom-made headsets for catheter exteriorization were described before [27-29]. Briefly, IP and HPV catheters both consisted of 20 cm silicone tubing [Dow Corning, Midland, MI; inner diameter (ID) x outer diameter (OD), 0.51 x 0.91 mm] connected to an L-shaped 20-G syringe. The connections were shielded with two 3 mm (ID x OD, 0.76 x 1.65 mm) and 2.2 cm (ID x OD, 1.02 x 2.18 mm) long pieces of silicon tubing as inner and outer layers, respectively. The catheters were sewn to a 2.5 x 3 cm oval of polypropylene surgical mesh (Marlex; Bard Implants, Billerica, MA) to improve adhesion to skin and fascia.

For infection prophylaxis and analgesia, rats were pretreated with 4 mg/kg trimethoprim/20 mg/kg sulfadoxine (Borgal 24%; Intervet/Shering-Plough Animal Health, Kenilworth, NJ) subcutaneously (SC) a few hours before surgery. Atropine (0.05 mg/kg; Sintetica, Mendrisio, Switzerland) was injected SC immediately prior to surgery. Rats were anesthetized by IP injection (1.2 ml/kg) of a mix of 80 mg/kg ketamine (Ketasol-100; Dr. E. Gräub AG, Bern, Switzerland), 4.0 mg/kg xylazine (Rompun; Bayer, Leverkusen, Germany) and 0.1 mg/kg acepromazine (Ozzano Emilia, Dietlikon, Switzerland). Catheter headsets were led SC from 2 cm midline interscapular incisions to puncture wounds 1 cm rostral to the incision, exteriorized and occluded with a stainless steel obturator until infusions or blood samplings took place. The proximal ends of the catheters were led SC from the neck to 4 cm midline laparotomies and through a puncture hole in the abdominal wall. IP catheters ended in the peritoneal cavity and were anchored on the left side of the abdominal wall with silk sutures. HPV catheters were inserted into the ileocolic vein, advanced into the HPV so that they ended 1 to 2 mm distal to the gastroduodenal vein, and anchored to the inside of the abdominal wall with silk suture (Silkam, 3/0; Braun, Tuttlingen, Germany) and histoacryl (Aesculap AG, Tuttlingen, Germany). The abdominal muscle wall and skin were closed with resorbable sutures (3-0 and 5-0 Vicryl, respectively; Ethicon, Norderstedt, Germany).

Four mg/kg trimethoprim/20 mg/kg sulfadoxine (Borgal 24%) and 5 mg/kg carprofen (Rimadyl; E. Gräub, Bern, Switzerland) were injected SC once per day for 2 days after surgery. IP catheters were flushed every 2-3 days with 0.5 ml 0.9% sterile saline, HPV catheters were flushed daily, first with 0.13 ml 0.9% sterile saline followed by 50% glycerol and 200 IU heparin/ml 0.9% sterile saline to prevent blood clotting. HPV catheterized rats remained in the experiment as long as blood could be collected from the catheter. Five rats had eventually to be excluded from the blood collection experiment because of catheter patency problems, resulting in n = 11 for analysis.

### Food intake

Starting 10 days after surgery, baseline food intake was assessed for several days. Just prior to dark onset, food cups were closed and refilled and reopened when all animals were infused (~15 min). At dark onset, animals were taken out of their cages, experimental solutions were infused manually over 30 sec and IP catheters flushed with 250 µl of 0.9% sterile saline to clear remaining solutions. To test for a possible dose-dependent effect, all animals (n = 23) randomly received either 10, 20, 40 or 80 mg/kg BW Wy-14643 dissolved in DMSO: Saline 70:30 (1 ml/kg) or vehicle (DMSO: Saline 70:30) alone through their IP catheters. The whole experiment consisted of five test days with one intervening day between them. For this experiment, rats were housed in 2 separate rooms and adjusted to staggered light cycles (dark onset at 0800 or 0900 h, respectively) to allow for staggered infusions on the same day. Cumulative food intake was measured for various times up to 48 h after infusion using custom-designed software (LabX pro balance, Mettler, Toledo, Greifensee, Switzerland). Meals were defined as ≥ 0.3 g food removals with ≥ 15 min intervals between them (inter-meal interval).

In a subsequent experiment rats received 40 mg/kg BW Wy-14643 dissolved in DMSO: Saline 70:30 (1 ml/kg BW) or vehicle alone in random order. The two trials of this within-subjects design were separated by one intervening day. Food intake was measured for 12 h and meal patterns were analyzed.

### Conditioned taste avoidance (CTA)

Twenty-two experimentally naïve rats were adapted to a 22 h daily water-deprivation schedule for 5 days, with 2 h water access at the end of the light phase (0600-0800 h). Two locations of water access were randomized during the adaptation period. Food was available *ad libitum*. On the last adaptation day, water intake was measured after 30 min and at 2 h, and rats were divided into three groups with roughly equal water intakes. On the conditioning day, rats were offered a novel 0.125% saccharin solution for the first 30 min of fluid access randomized between two locations in the cage. The saccharin solution was then removed and the rats were infused IP with either WY 14643 (40 mg/kg/1ml of DMSO/Saline 70:30, n = 10), vehicle (n = 9) or LiCl (60 mg/kg in 9.4 ml/kg H_2_O, n = 3) as a positive control, and water was offered for 90 min and again the next day for 2 h (intervening day). The test day was the following day. Both, 0.125% saccharin solution and water were offered at randomized locations, and 30 min intakes were recorded in the two-bottle preference test.

### Plasma metabolites

Sixteen rats with IP and HPV catheters weighing 350-380 g were maintained on the HFD for three weeks. Blood was collected on two days separated by two intervening days. On experimental days only animals were food deprived for approximately 10 h prior to infusions, the catheters were checked for patency, flushed with saline and the catheter tips filled with 120 µl tri-sodium citrate 7.5% (BHD Chemicals Ltd, Poole, UK) approximately 1.5 h prior to dark onset. Then the rats were left undisturbed in their cages for approximately 40 min before blood withdrawal began (baseline). Twenty min before dark onset rats received a 3 g HFD test meal that all animals ate within 10 min. Just before dark onset rats were IP infused with either Wy-14643 (40 mg/kg/ml DMSO: saline 70:30) or vehicle and HPV blood samples (450 µl each) were taken from each animal at 35 and 70 min after infusion. When blood was taken, the first 100 µl were returned to the animals after sampling 450 µl of blood into syringes filled with 1.5 mg/ml EDTA (Titriplex 3, Merck, Darmstadt, Germany). Blood was immediately transferred into Eppendorf tubes (Microvette CB 300, Sarstedt, Nümbrecht, Germany) containing 500 KIU/ml Aprotinin (Sigma-Aldrich, Buchs, Switzerland), kept on ice for a maximum of 20 min, and centrifuged (10 min, 10.000 rpm, 4 °C). Plasma was stored at -80 °C for later analysis of non-esterified fatty acids (NEFA) and β-hydroxybutyrate (BHB). To compensate for the withdrawn blood volume, each rat was infused immediately after the final blood sampling with 1 ml of freshly taken (1–2 h before use) donor blood preserved in ACD solution (2.2% sodium citrate, 0.8% citric acid monohydrate, 2.45% glucose monohydrate; Cantonal Pharmacy, Zurich, Switzerland). Blood from donor rats was taken by cardiac puncture. Similar blood transfusions appeared to not affect plasma levels of glucose, NEFA, insulin and other endocrine parameters in previous studies [30-32]. Plasma levels of NEFA and BHB were determined using standard colorimetric and enzymatic methods adapted for the Cobas, Mira auto-analyzer ( [33], Hoffman LaRoche, Basel, Switzerland).

### Indirect calorimetry

Sixteen rats weighing 260-280 g were individually housed in indirect calorimetry cages under a 12: 12h dark/light cycle (lights off at 0900 h) in a climate-controlled room (22 ± 2 °C and 60% relative humidity). The animals were adapted to housing and diet conditions for at least one week prior to the start of the experiment. The recordings of body temperature, physical activity and gas exchange were performed as previously described [34]. Seven rats were implanted with IP catheters, and small animal sensors (DSI PhysioTel TA-F40, Data Sciences International, St. Paul, MN, USA) were attached to the abdominal wall to record body temperature and locomotor activity. Treatments (Vehicle or Wy-14643) were given randomly 15 min prior to the dark onset in a within-subjects crossover design on two experimental days with two intervening days. The recordings continued for 12 hours. Changes in oxygen and carbon dioxide concentrations were recorded from each cage every 30 second by an online computerized data acquisition system (PhysioPlot Version 1.80, Integra ME Version 2.21; AccuScan Instrument Inc.).

### Western blots

The intestinal mucosa of 22 rats weighing 460-500 g was scraped off from the small intestine (duodenum, and jejunum) as described previously [35], 1 h after infusion of vehicle or Wy-14643. Whole proteins were extracted from the liver and the intestinal mucosa using lysis buffer (1% Triton 100 in PBS containing protease and phosphatase inhibitors) and the protein content was estimated using Bio-Rad protein assay (Bio-Rad DC™ protein assay kit). Briefly, proteins (25 to 50 µg) were loaded in a 7% or 10% SDS-PAGE gel and then transferred onto a nitrocellulose membrane. Membranes were blocked with 5% (w/v) milk in Tris buffered saline (TBS) for 1 h at room temperature (RT). Membranes were incubated with the primary antibody against β-actin (clone AC-74, Sigma-Aldrich, A2228); mitochondrial 3-hydroxy-3-methylglutaryl-CoA synthase (HMG-CoAS2) polyclonal antibody (Aviva systems biology, ARP41562_T100); rat CPT 1A or CPT 2 [36] overnight at 4 °C, followed by the appropriate secondary antibody for 1 h at RT. Protein bands were visualized by enhanced chemiluminescence (ECL) using Image Quant LAS 400 mini system.

### Lipid droplets

Nineteen rats weighing 430-480 g were infused IP with either Wy-14643 (40 mg/kg/1ml of DMSO/Saline 70:30, n = 10) or vehicle (n = 9) at dark onset. About 40-60 min after the infusion rats were anesthetized with an overdose of pentobarbital-Na (100 mg/kg, IP, Kantonsapotheke Zürich, Switzerland). The thorax and abdominal cavity were opened, the inferior vena cava clamped below the liver, the right ventricle cut and livers were perfused with ^~^50 ml of 0.1 M phosphate buffered saline (PBS) at 4 °C. The right upper liver lobe and three approximately 0.5 cm long samples of the duodenum, jejunum and ileum were removed and snap frozen in O.C.T. embedding matrix (Biosystem, Nunningen, Switzerland). Samples were sectioned in the sagittal plane on a cryostat at a thickness of 7 µm and allowed to dry for 4 h before storing at -20 °C until processing. Slides were then fixed with 4% formaldehyde in 1 x PBS for 1 h, rinsed 3 x with PBS and 1 x with sterile H_2_O. Nuclei, cytosol and lipid droplets were then stained with Hoechst (1 : 500, Invitrogen, Basel, Switzerland), Syto60 (1 : 1000, Invitrogen, Basel, Switzerland) and Bodipy (1 : 1000, Invitrogen, Basel, Switzerland) in sterile H_2_O for 1 h. Slides were rinsed with sterile H_2_O and incubated in 1.5% fat free bovine serum albumin (Sigma-Aldrich, Buchs, Switzerland) for 1 min, rinsed with PBS, dispended in mounting medium (Vectashield, Vector Laboratories, Peterborough, UK), and cover slipped thereafter. From a large number of samples pictures were taken randomly with an Operetta TM microscope (PerkinElmer, Waltham, MA, USA) within 24 h after staining. Pictures that contained the intestinal muscle wall were excluded from further analysis. A minimum of ten pictures was analyzed for the liver and intestinal mucosa per rat. Pictures were analyzed automatically for number of and area covered by lipid droplets [expressed as area per pixel (px^2^)] within the cytosol using Harmony 3.0 (PerkinElmer, Waltham, MA, USA).

### Statistical analysis

Data were analyzed using non-parametrical statistical analysis as appropriate. Pair-wise comparisons were analyzed with Wilcoxon Signed-Rank and Mann-Whitney U-tests for dependent and independent samples, respectively. Data for cumulative food intake and meal patterns were analyzed using paired *t*-tests (data presented as means ± SEM) or Wilcoxon Signed-Rank (data presented as median with 25th/75th percentiles). Separate analyses were performed at each time point, and data were corrected by means of series for missing values. Non-normally distributed data were transformed using logarithmic or square-root transformations to improve normality or else non-parametric tests were used. Data from first meal size were not normally distributed even after using various transformations such as square roots, logarithmic and inverse transforms. The normal distribution was assessed by looking at the histogram of frequency distributions or probability (*P*) value output from the SPSS (SPSS Statistical package, version 17.0). Differences between means were considered significant, if *P* < 0.05.

## Results

### Wy-14643 reduced food intake in HFD-fed rats

**Figure 1:**
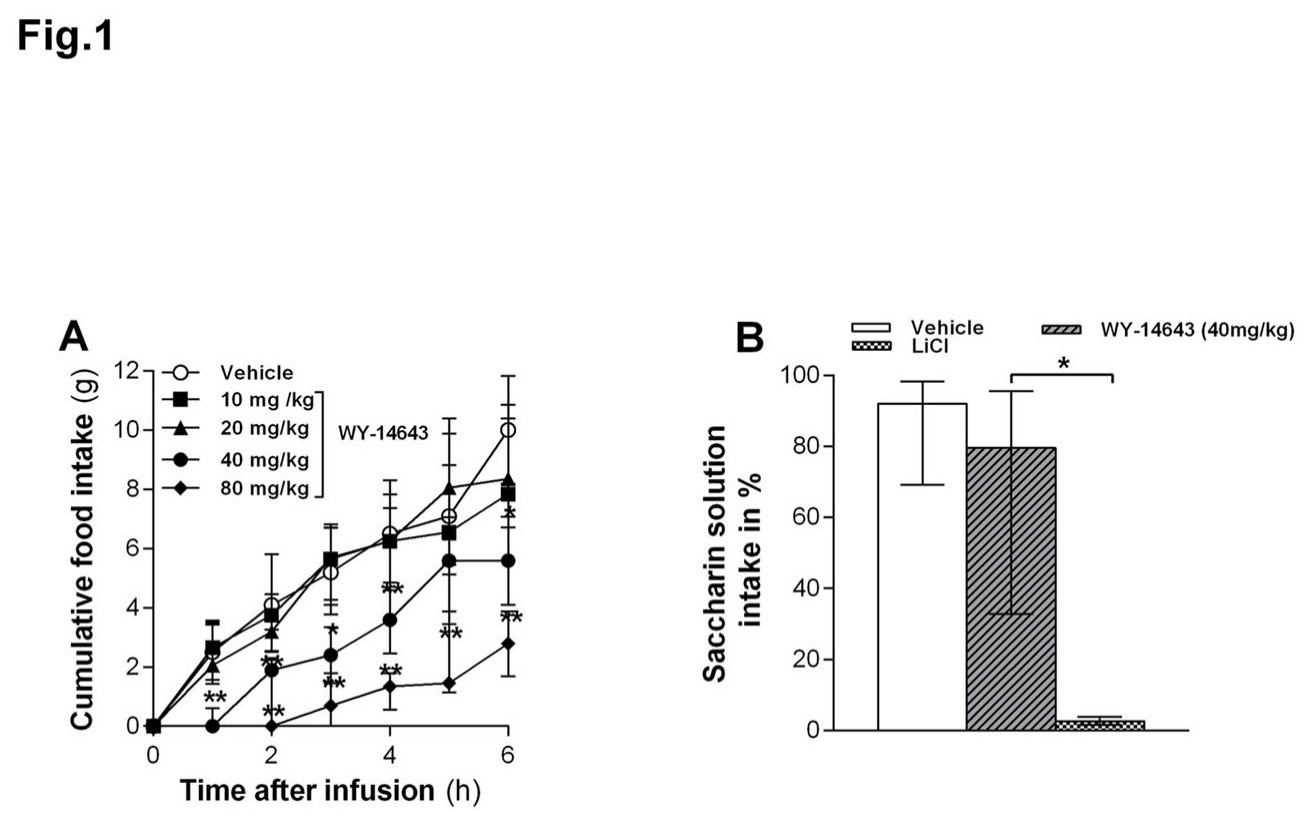
Effect of intraperitoneally (IP) injected Wy-14643 on food intake. (**A**) IP injected Wy-14643 (10, 20, 40 or 80 mg/kg/ml) dose-dependently reduced cumulative food intake (FI) in ad libitum HFD-fed rats (n=23). * Less than vehicle (*P* < 0.05), ** less than vehicle (*P* < 0.01), Mann-Whitney U test. (**B**) IP administered Wy-14643 (40 mg/kg/ml, n=10) did not reduce saccharin solution intake (% of total fluid intake) vs. water in a two-bottle preference test, whereas LiCl (60 mg/kg in 9.4 ml/kg H_2_O, n = 3) did. The height of each bar represents the median and the top and bottom of each bar shows 75th and 25th percentile respectively. * Significantly different from vehicle (*P* = 0.05).

IP injected 80 and 40 mg/kg BW Wy-14643 reduced food intake at all time points during six hours when compared with vehicle, whereas the 10 and 20 mg/kg doses did not (*P*s < 0.05, Figure 1A). The hypophagia after 40 mg/kg was still present after 9 hours and the effect of 80 mg/kg was extremely strong in the beginning (the animals basically stopped eating) and had still not fully recovered at 48 h after administration when food intake in the other groups did not differ anymore (data not shown). Therefore, the remaining experiments were conducted using the lowest dose that reliably and transiently inhibited eating under our conditions (40 mg/kg).

### Wy-14643 (40 mg/kg) did not induce a CTA

IP Wy-14643 (40 mg/kg) did not induce a significant decrease in saccharine solution preference compared to vehicle (*P > 0.05*, Figure 1B). As expected, LiCl did induce a strong avoidance. These findings suggest that IP Wy-14643 (40 mg/kg) does not induce a CTA.

### Wy-14643 increased mainly the latency to eat

**Figure 2:**
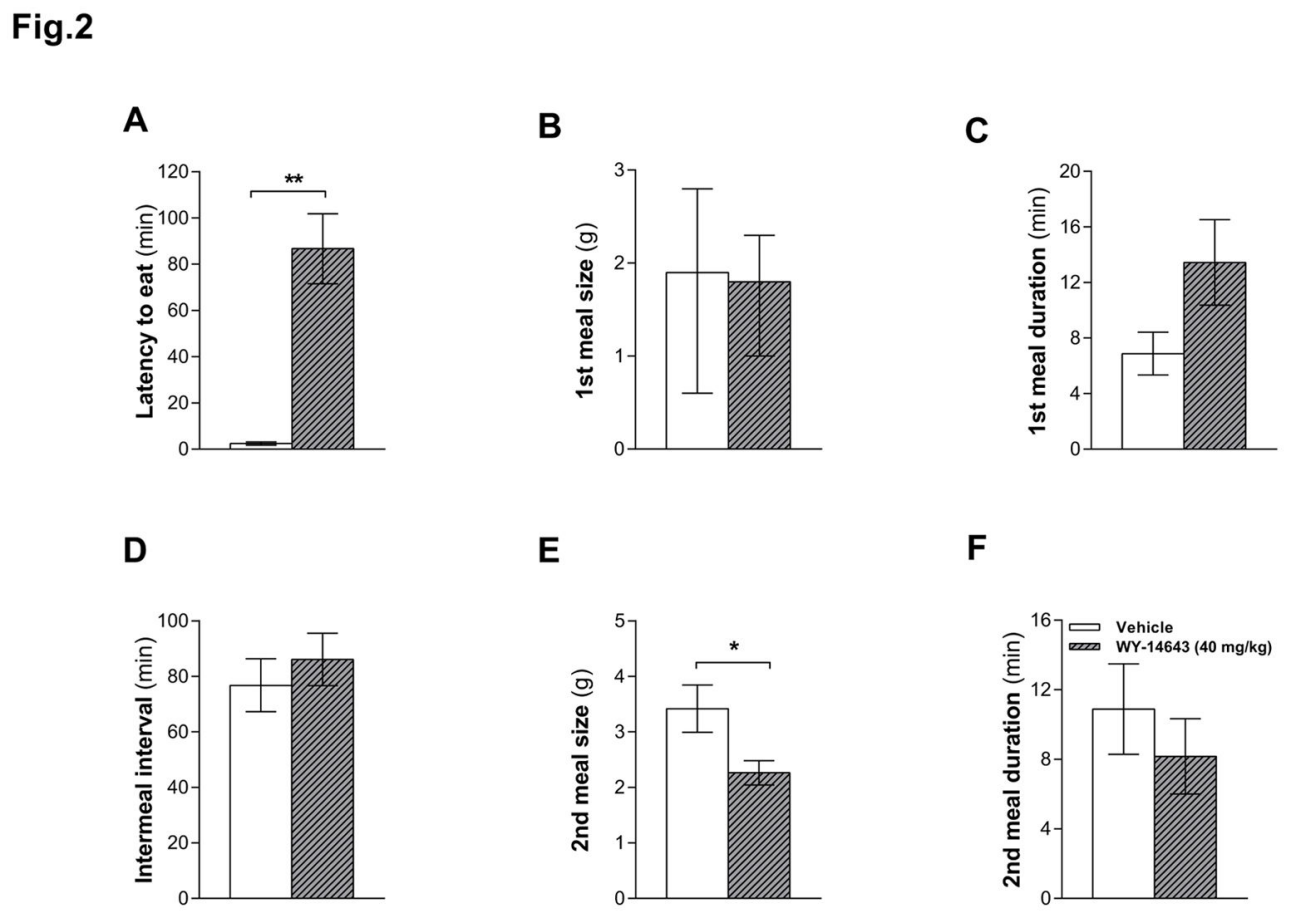
Effect of intraperitoneally (IP) injected Wy-14643 on meal pattern. IP administered Wy-14643 (40 mg/kg/ml) on meal patterns in ad libitum HFD-fed rats (n = 23). Wy-14643 increased the latency of the first meal (**A**) and decreased the second meal size (**E**), but did not significantly affect first meal size and duration (**B**, **C**), intermeal interval (**D**) and second meal duration (**F**). Data for first meal size are presented as median with percentiles and were analyzed with Wilcoxon Signed Ranks Test. Results for the other parameters (latency to eat, first meal duration, second meal size and duration) are presented as means ± SEM and were analyzed using a paired *t*-test. ** Significant difference (*P* < 0.01) compared to vehicle.

IP Wy-14643 (40 mg/kg) again reduced food intake compared to vehicle (2 h cumulative food intake was 1.6 ± 0.4 and 4.5 ± 0.8 g (mean ± SEM) after Wy-14643 and vehicle, respectively, *P* < 0.05) and did so mainly by increasing the latency to eat (*P* < 0.05, Figure 2A). Wy-14643 did not affect first meal size (Figure 2B) or the duration of the first intermeal interval (IMI, Figure 2D); it tended to increase first meal duration, but this effect did not reach statistical significance (Figure 2C). Likewise, first meal eating rate was not significantly affected (0.24 ± 0.054 and 0.32 ± 0.048 g/min (mean ± SEM) for Wy-14643 and vehicle, respectively (*P* > 0.05)). Wy-14643 also reduced second meal size, but not duration (Figure 2E and 2F) and, hence, did not affect the second meal eating rate either (0.37± 0.033 and 0.34 ± 0.037 g/min (mean ± SEM) for Wy-14643 and vehicle, respectively (*P* > 0.05)). Also, Wy-14643 did not affect the size and duration of subsequent meals or the duration of subsequent IMIs (data not shown).

### Wy-14643 increased circulating BHB in HFD-fed rats

**Figure 3:**
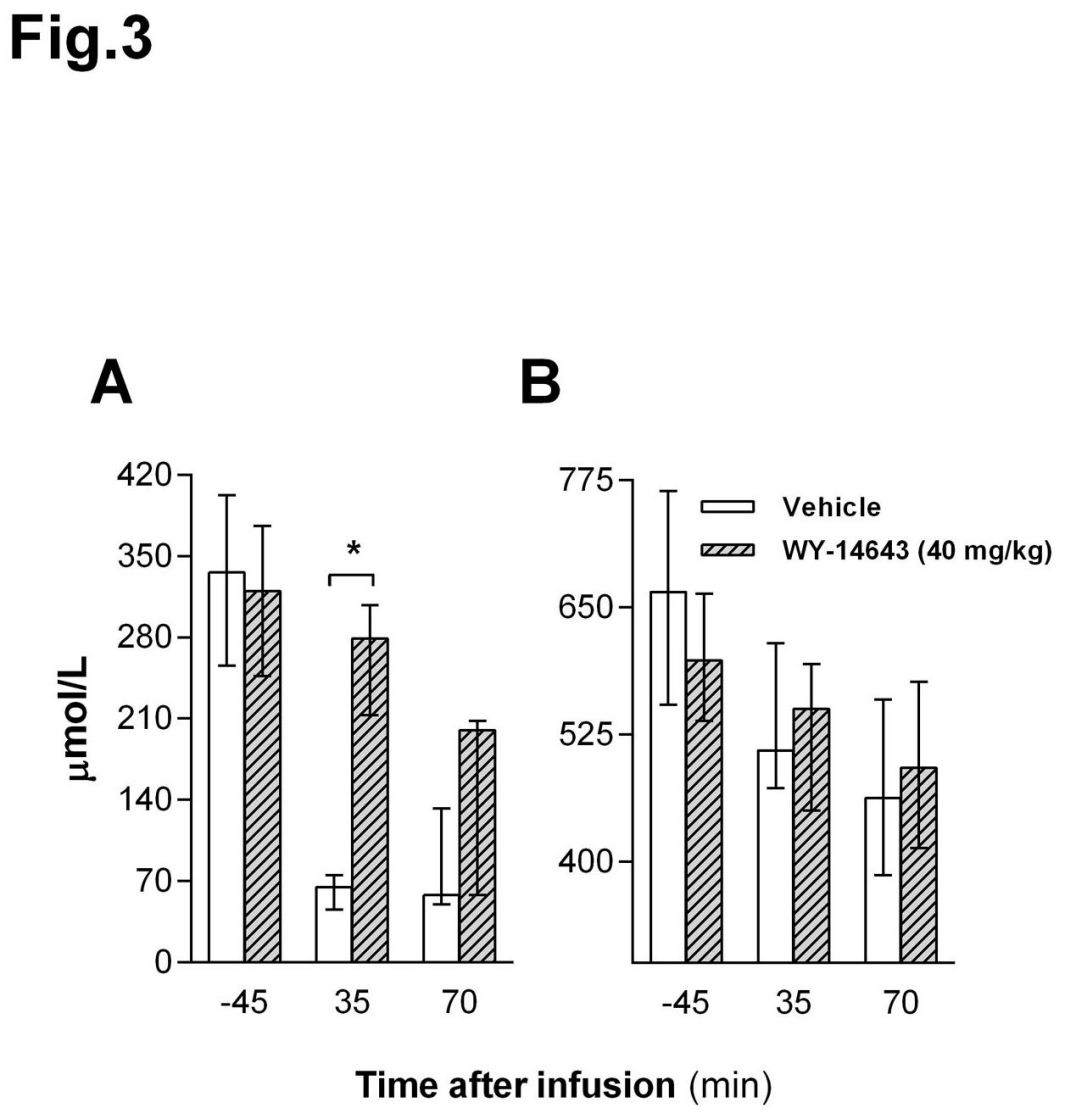
Effects of intraperitoneal (IP) administration of Wy-14643 on plasma metabolites. β-hydroxybutyrate (BHB) (**A**) and non-esterified fatty acids (NEFA) (**B**) levels were assessed 45 min before (baseline), 35 and 70 min after treatment (Wy-14643, 40 mg/kg/ml) and 3g HFD test meal. ** Significant difference compared to vehicle (*P* < 0.01; Wilcoxon Signed Ranks Test, data are presented as median with 25th/75th percentiles.

Given the stimulatory effect of PPAR-α activation on FAO, we measured some metabolite levels in HPV plasma after IP injection of Wy-14643 or vehicle and a 3 g HFD test meal. Interestingly, Wy-14643 (40 mg/kg BW) transiently increased plasma BHB compared to vehicle 35 min after administration (*P* < 0.05, Figure 3A), whereas there was no significant effect on circulating NEFA levels (Figure 3B).

### Wy-14643 reduced RQ and EE in HFD-fed rats

**Figure 4:**
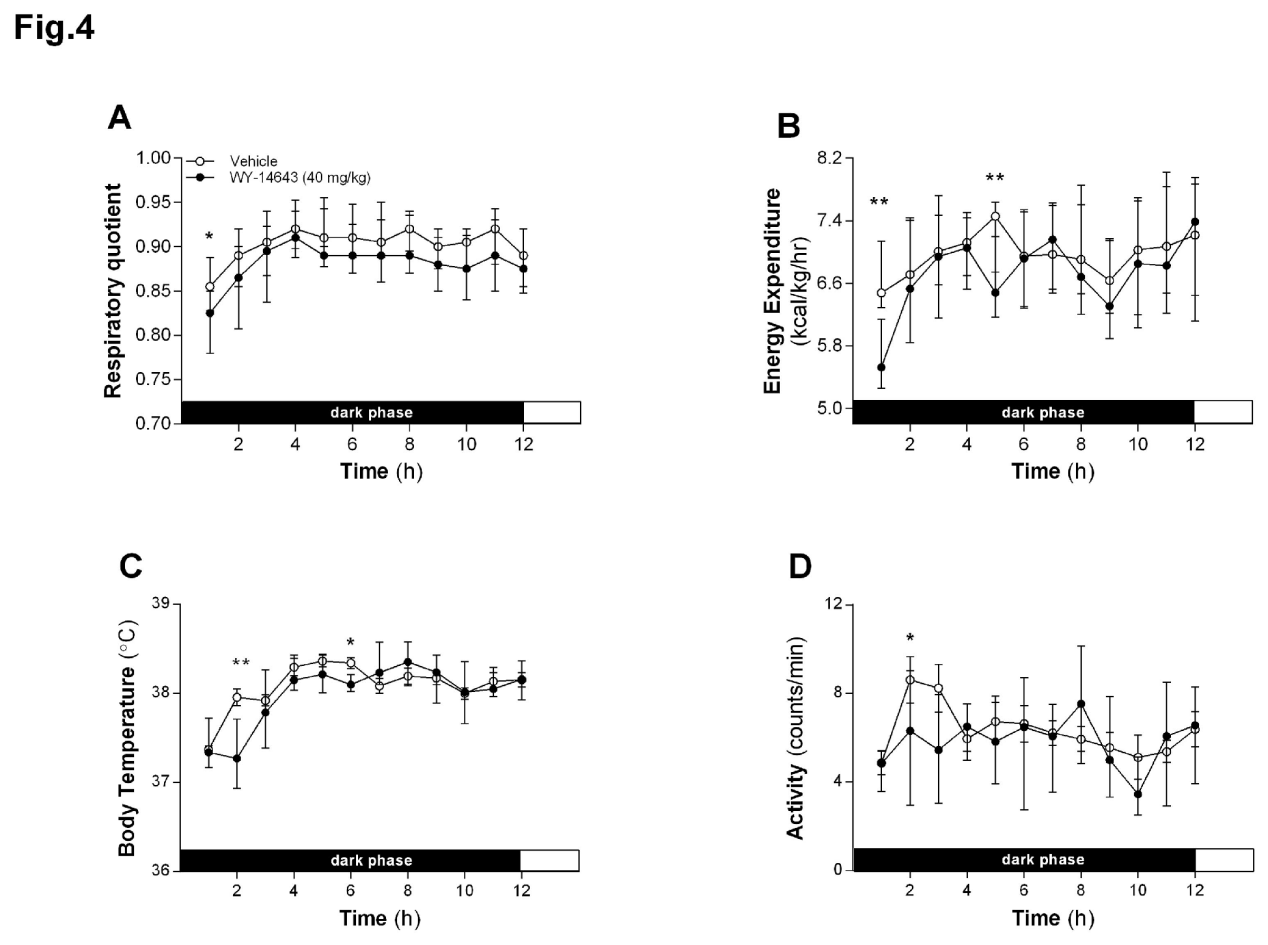
Effect of intraperitoneal (IP) administration of Wy-14643 on energy homeostasis. Respiratory quotient (**A**), spontaneous locomotor activity (**B**), energy expenditure and body temperature (**C**, **D**) were assessed during 12 h following IP administration of Wy-14643 (40 mg/kg/ml). * and** significantly different from vehicle (*P < 0.05, **P < 0.01; Wilcoxon Signed Ranks Test, data presented as median with 25th/75th percentiles.

IP Wy-14643 (40 mg/kg BW) reduced the RQ in the first hour post-infusion (*P* < 0.05, Figure 4A), indicating a metabolic shift towards FAO. Wy-14643 also reduced EE at various time points between 1 and 5 h after infusion (*P* < 0.01, Figure 4B). This effect was associated with a reduced physical activity at 2 h (Figure 4D) and hypothermia at 2 and 6 h post-infusion (Figure 4C).

### Wy-14643 induced expression of CPT-1A and HMG-CoAS2 in the jejunum of HFD-fed rats

**Figure 5:**
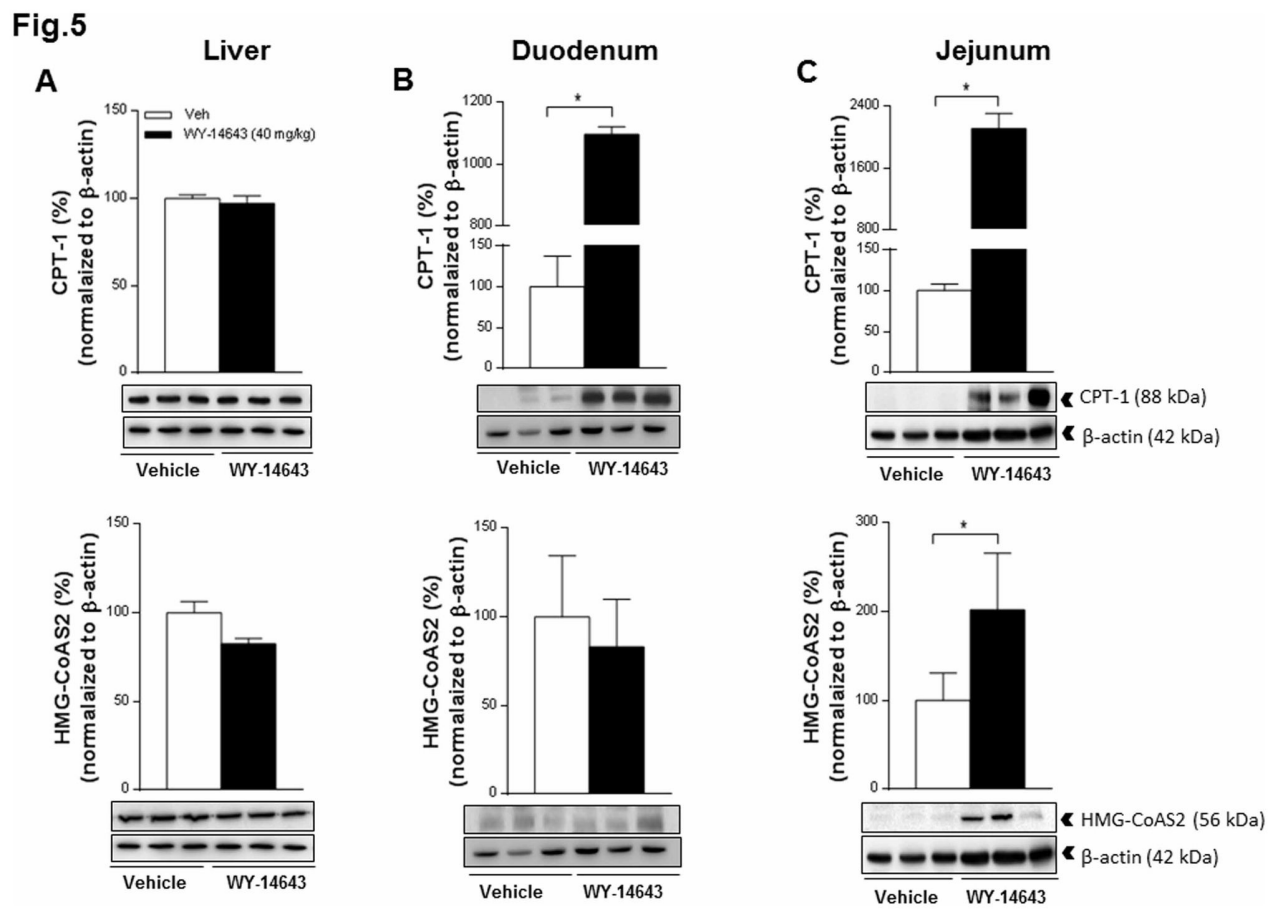
Effect of intraperitoneal (IP) administration of Wy-14643 on the protein expression pattern. Carnitine palmitoyltransferase 1A (CPT 1A) (top), mitochondrial 3-hydroxy-3-methylglutaryl-CoA synthase (HMG-CoAS2) (bottom) and β-actin in the liver (A), the duodenum (B) and the jejunum (C) were analyzed by Western blotting 1h after IP administration of Wy-14643 (40 mg/kg/ml). Panels below the graphs show representative blots; upper panels show quantification of protein levels after normalization to β-actin. Data are presented relative to the vehicle (100%) and each bar represent median with 25th/75th percentiles (* *P*<0.05; Mann-Whitney U test).

IP Wy-14643 (40 mg/kg) induced a strong protein expression of CPT 1A in the duodenum and the jejunum, but not in the liver. IP Wy-14643 did not affect the expression of CPT 2 in the intestine or liver (data not shown). Moreover, Wy-14643 induced the expression of the mitochondrial HMG-CoAS2 in the jejunum, but not in the duodenum or liver (Figure 5).

### Lipid droplets in the small intestine

**Figure 6:**
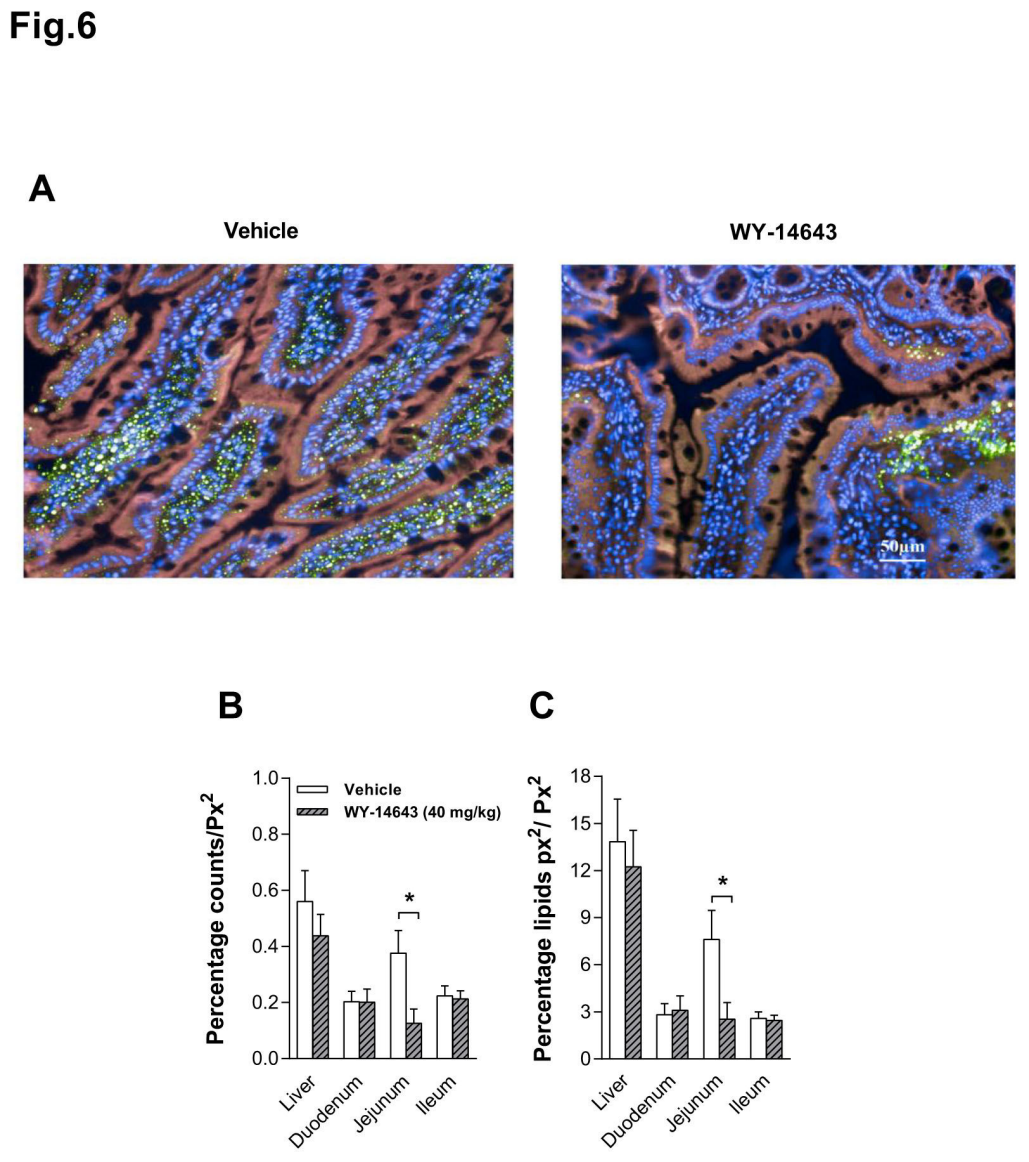
Effects of intraperitoneal (IP) administration of Wy-14643 on lipid droplets. The picture shows a representative photographic representation of lipid droplets (green) within the cytosol (pink) of the intestinal mucosa of the jejunum (**A**) of a rat treated with IP Wy-14643 (40 mg/kg/ml, right) or vehicle (1 ml/kg, DMSO: saline 70:30, left) (Color: blue = nuclei). Each bar represent median with 25th/75th percentiles of 19 rat of the number of lipid droplets (**B**) within the cytosol [cytosol expressed as area per pixel (px^2^)] and the area occupied by lipid droplets (**C**) of the liver and intestinal mucosa in percent. * Significantly different from vehicle (*P*< 0.05).

IP Wy-14643 (40 mg/kg) decreased the number and the area of lipid droplets in the jejunum of HFD-fed rats (median (Mdn) = 0.065 and 1.58 respectively, *P*s < 0.05, Figure 6A), but had no effect on both parameters in the liver (Mdn = 0.36 and 9.12), duodenum (Mdn = 0.155 and 2.32) and ileum (Mdn = 0.244 and 2.72) (Figure 6B and 6C).

## Discussion

This study examined the eating-inhibitory effect of the peroxisome proliferator-activated receptor alpha (PPAR-α) agonist Wy-14643 and tried to identify a possible mechanism of action. Intraperitoneally (IP) administered Wy-14643 (40 mg/kg BW) reduced food intake in HFD-fed rats mainly by increasing the first meal latency and without inducing a conditioned taste avoidance. In parallel to its eating-inhibitory effect, Wy-14643 decreased the respiratory quotient (RQ) and increased the expression of carnitine palmitoyltransferase-1A (CPT 1A) protein in the duodenum and the jejunum, but not in the liver. Furthermore, compared to vehicle Wy-14643 increased hepatic portal vein β-hydroxybutyrate (BHB) levels 35 min after administration without affecting circulating non-esterified fatty acids (NEFA), and induced the expression of the mitochondrial 3-hydroxy-3-methylglutaryl-coenzyme-A synthase (HMG-CoAS2) protein specifically in the jejunum. Together these findings suggest that stimulation of small intestinal FAO by Wy-14643 contributed in the inhibition of eating with HFD feeding.

The Wy-14643-induced reduction in food intake is consistent with previous findings in mice [22] and rats [9] and fits reports of reductions in food intake in response to administration of the PPAR-α agonists fenofibrate [14,37] or oleylethanolamide (OEA) [9,22,38]. Moreover, it has been shown that blocking the PPAR-α abrogated the satiety effect of linoleoylethanolamide (LEA), indicating that PPAR-α is involved in LEA’s eating-inhibitory effect [39]. Wy-14643 and OEA have been shown to delay eating onset without affecting first meal size and the duration of the subsequent inter-meal interval (IMI) [22,40] when animals were fed *ad libitum*. Similarly, in our study Wy-14643 administration in HFD-fed rats predominantly affected the latency to eat. The reduction of the second meal size does not contradict this interpretation; it only shows that Wy-14643 can also reduce meal size under some conditions. The major initial decrease in food intake, reflected in the shift of the cumulative intake curve to the right (Figure 1), was due to the increased latency to eat. At least a relative reduction of meal size also occurred in the first meal because otherwise a larger meal should have occurred after a latency of about 80 min than after a latency of about 3 min [41]. In general, the meal pattern findings fit other studies showing that modulating lipid metabolism via an inhibition of FAO using mercaptoacetate (MA) [42], or an inhibition of fatty acid synthesis and, perhaps, stimulating FAO using C75 [43], affects eating primarily by inducing changes in meal frequency without major effects on meal size. Together these findings support the hypothesis that FAO contributes to the maintenance of satiety between meals [42,44]. An enhancement or inhibition of FAO might therefore prolong or shorten the IMI, respectively.

We here report for the first time that Wy-14643 reduced the RQ in HFD-fed rats. This reduction was already evident during the first half hour after Wy-14643 administration, indicating an acute increase in whole body FAO in response to Wy-14643. The decrease in RQ occurred together with a transient decrease in body temperature and energy expenditure (EE). The indirect calorimetry data of course do not disclose where these effects originated and the possibility that liver and adipose tissues contributed to the observed changes in energy expenditure and reduction body weight gain cannot be excluded. The observed initial decrease in EE and body temperature in Wy-14643 treated rats may be related to the reduction in activity and food intake, and the concomitant reduction in the thermic effect of food.

To identify the organ/site where the increase in FAO took place, we analyzed the protein expression pattern of key enzymes involved in mitochondrial FAO and ketogenesis in the liver and the small intestine, two organs known to have a high expression level of PPAR-α and to be key players in lipid metabolism [18,20]. We found a clear induction of CPT 1A expression in the jejunum and duodenum, and of HMG-CoAS2 expression in the jejunum, but not in the liver. The lack of an effect in the liver is surprising, given that PPAR-α agonists stimulated the hepatic gene expression of these enzymes in previous studies [14,45,46]. It has been shown, however, that in the case of mitochondrial HMG-CoA synthase for instance, the protein expression level does not reflect the mRNA expression level in the liver of starved rats [47]. Thus, differences in the experimental conditions may lead to differential changes in the mRNA and protein levels for the mitochondrial synthase. Our data therefore do not exclude that Wy-14643 stimulated FAO and ketogenesis also in the liver without changing the protein expression level of the pertinent enzymes, but in any case the findings indicate that Wy-14643 also affected FAO in the small intestine, and presumably more so than in the liver. CPT 1A and HMG-CoAS2 play major roles in the control of intestinal FAO and ketogenesis during the suckling period, and their expression decreases after weaning [48]. PPAR-α can bind to a specific DNA sequence, called peroxisome proliferator response elements (PPRE) that regulates the expression of a wide range of enzymes implicated in FAO [12,13,21,49], and is located in the promoter of mitochondrial HMG-CoA synthase and some other genes [45,50,51]. The PPAR-α receptor is highly expressed in the duodenum and in jejunal villus cells [18]. In addition, morphological studies following Wy-14643 treatment indicate that PPAR-α activation increases the height of the villi [18]. Consistent with our findings, PPAR-α ligands (fenofibrate, clofibrate and Wy-14643) have been shown to increase the binding of PPAR-α to PPRE and to up-regulate the gene expression of several lipid catabolism enzymes including HMG-CoAS2 and CPT 1 in the small intestine, but not in the liver of HFD-fed mice [45,52-54]. Because the small intestine is the primary site exposed to dietary fats, where fat digestion and uptake occurs, the presence of PPAR-α in the small intestine has been suggested to be an important adaptive response for lipid absorptive capacity [14,18].

Several studies described the capacity of the intestine to oxidize fat and to produce ketone bodies even after weaning. Fat absorption occurs primarily in the jejunum, where dietary-derived long-chain fatty acids pass the brush border membrane mainly by diffusion. Once inside the cell, the fatty acids are reesterified to triglycerides, but they can also be stored in lipid droplets or be oxidized [14]. In fact, the liver isoform of the fatty acid binding protein that is expressed in jejunal enterocytes directs fatty acids towards oxidation [55]. Furthermore, it has been shown that intrajejunal infusion of diacylglycerol increases FAO specifically in the small intestine [56]. The observed induction of CPT 1A and HMG-CoAS2 protein expression by a PPAR-α activator specifically in the intestine and not in the liver is a novel finding, suggesting that the intestine is a major site of Wy-14643 action to stimulate FAO and ketogenesis. Given the major role of the jejunum in nutrient absorption with the ensuing high-energy requirements, and the jejunal FAO and ketogenesis capacity, it is probably not surprising to find that Wy-1643 induced protein expression of CPT 1 and HMG-CoAS2 in the jejunum, in particular under our conditions, i.e., with HFD feeding.

The decrease in lipid droplet content after IP Wy-14643 administration in the jejunum suggests that some of the fatty acids that fuel the stimulation of intestinal FAO and ketogenesis by Wy-14643 are derived from a mobilization of temporarily stored triglycerides in enterocytes. In line with our finding, Uchida and colleagues [14] showed that activation of PPAR-α by fenofibrate stimulated FAO in the jejunum and reduced triglyceride storage in the enterocytes of mice. In fact, fenofibrate’s effect on circulating triglycerides appeared to be mediated by a reduction of triglyceride secretion into intestinal lymph, resulting from an increase in intestinal FAO in HFD-fed mice [14].

Interestingly, compared to vehicle, Wy-14643 increased hepatic portal vein (HPV) BHB levels 35 min after administration without affecting circulating NEFA. Usually there is a close positive relationship between circulating NEFA and hepatic ketogenesis, and access to food (carbohydrate) induces a decrease in both. This is primarily due to an increase in insulin release during eating, which blocks adipose tissue lipolysis, stimulates hepatic lipogenesis and thus inhibits hepatic FAO and ketogenesis [57]. Therefore, the observed eating-related relative increase in HPV BHB together with the lack of a rise in NEFA (in fact, NEFA appeared to decrease as should be expected upon feeding, but this difference was not statistically significant), and the increase in jejunal expression of HMG-CoAS2, all indicate that the relative increase in circulating BHB originated primarily from the intestine and not from the liver.

Whether the transiently increased BHB level after Wy-14643 administration itself has a signaling function in the inhibition of eating remains unclear. Such an effect is feasible, however, because ketone bodies have been shown to reduce food intake after peripheral [58] and central [59] administration and may be involved in the physiological control of eating [60]. In any case, the inhibition of eating occurred at the same time as the BHB release, the shift in the respiratory quotient towards FAO, the induction of the expression of the key enzymes involved in FAO and ketogenesis and the reduction in the level of lipid droplets in the jejunum after Wy-14643 administration. This suggests that the eating-inhibitory of Wy-14643 effect is related to these changes and may be a result of the stimulation of intestinal FAO.

In sum, we found that Wy-14643 reduced food intake after IP administration by increasing first meal latency without inducing a conditioned taste avoidance. Therefore, Wy-14643 predominantly enhanced satiety. The most probable mechanism for this inhibition of eating is the activation of intestinal FAO and, perhaps, ketogenesis through a PPAR-α-mediated up-regulation of the pertinent key enzymes. Our findings therefore suggest a role of intestinal FAO in the control of eating and identify intestinal fat handling as a potential target in the treatment of obesity. Given that the data are only correlative, however, further studies are necessary to critically examine a possible causal relationship.
